# Supplementary figures and images for: Dual specificity phosphatase 7 drives the formation of cardiac mesoderm in mouse embryonic stem cells
Source: PLoS One. 2022 Oct 13;17(10):e0275860. doi: 10.1371/journal.pone.0275860 (PMC9560500; doi:10.1371/journal.pone.0275860)

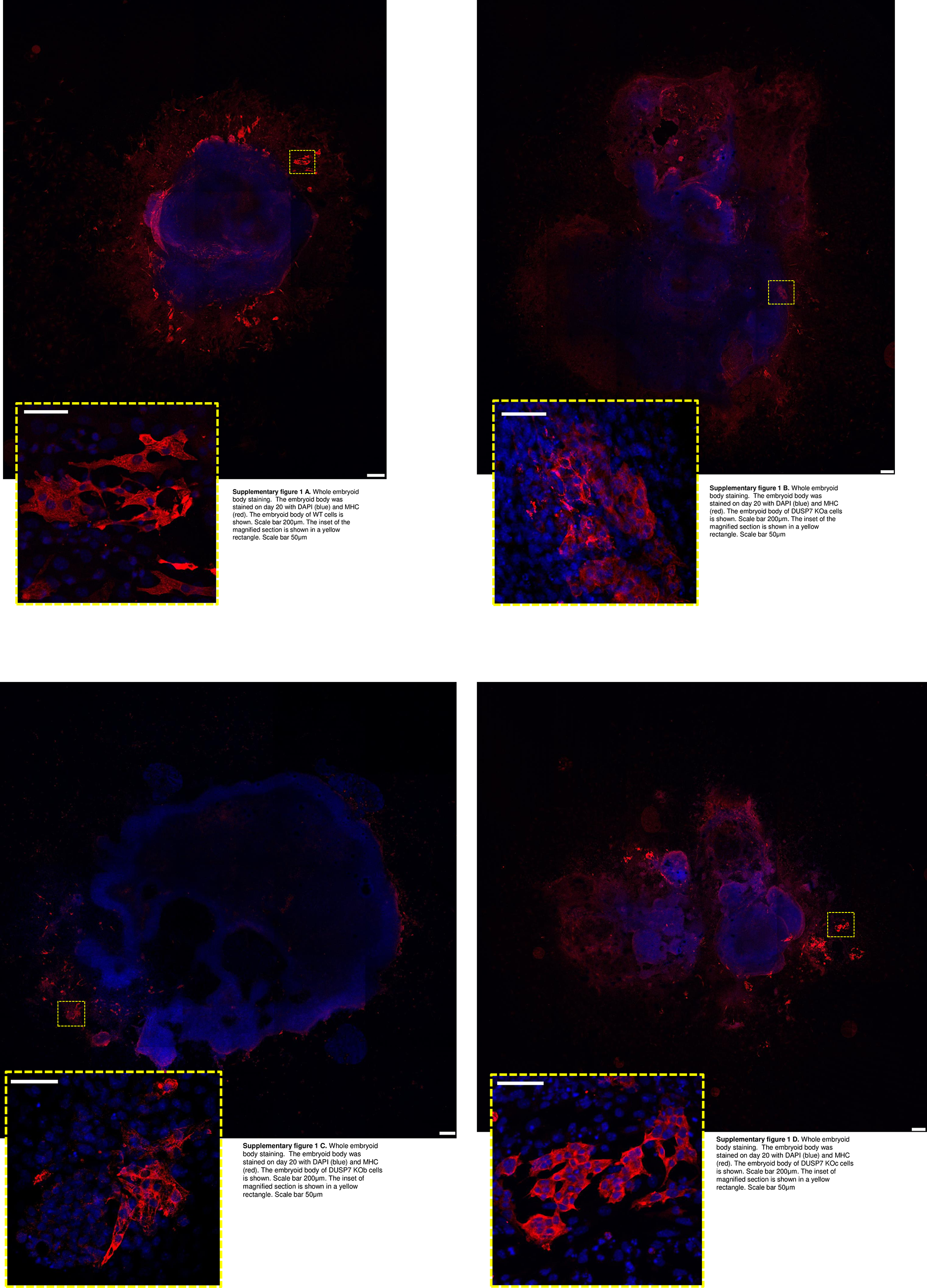

Supplement: S1 Fig — A: Whole embryoid body staining of WT cells, B: Whole embryoid body staining of DUSP7 KOa cells, C: Whole embryoid body staining of DUSP7 KOb cells, D: Whole embryoid body staining of DUSP7 KOc cells. (TIF) [file pone.0275860.s001.tif]

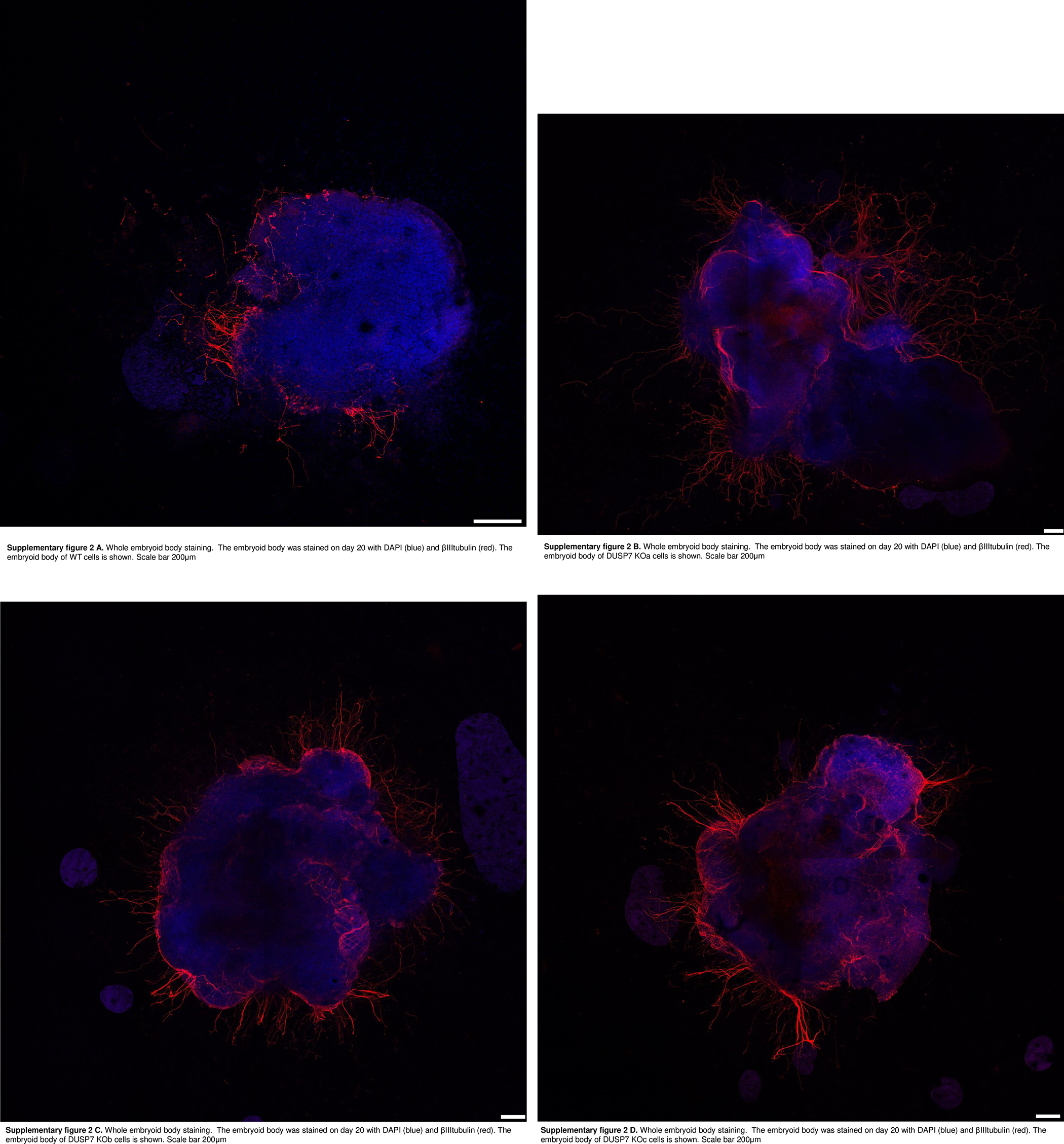

Supplement: S2 Fig — A: Whole embryoid body staining of WT cells, B: Whole embryoid body staining of DUSP7 KOa cells, C: Whole embryoid body staining of DUSP7 KOb cells, D: Whole embryoid body staining of DUSP7 KOc cells. (TIF) [file pone.0275860.s002.tif]

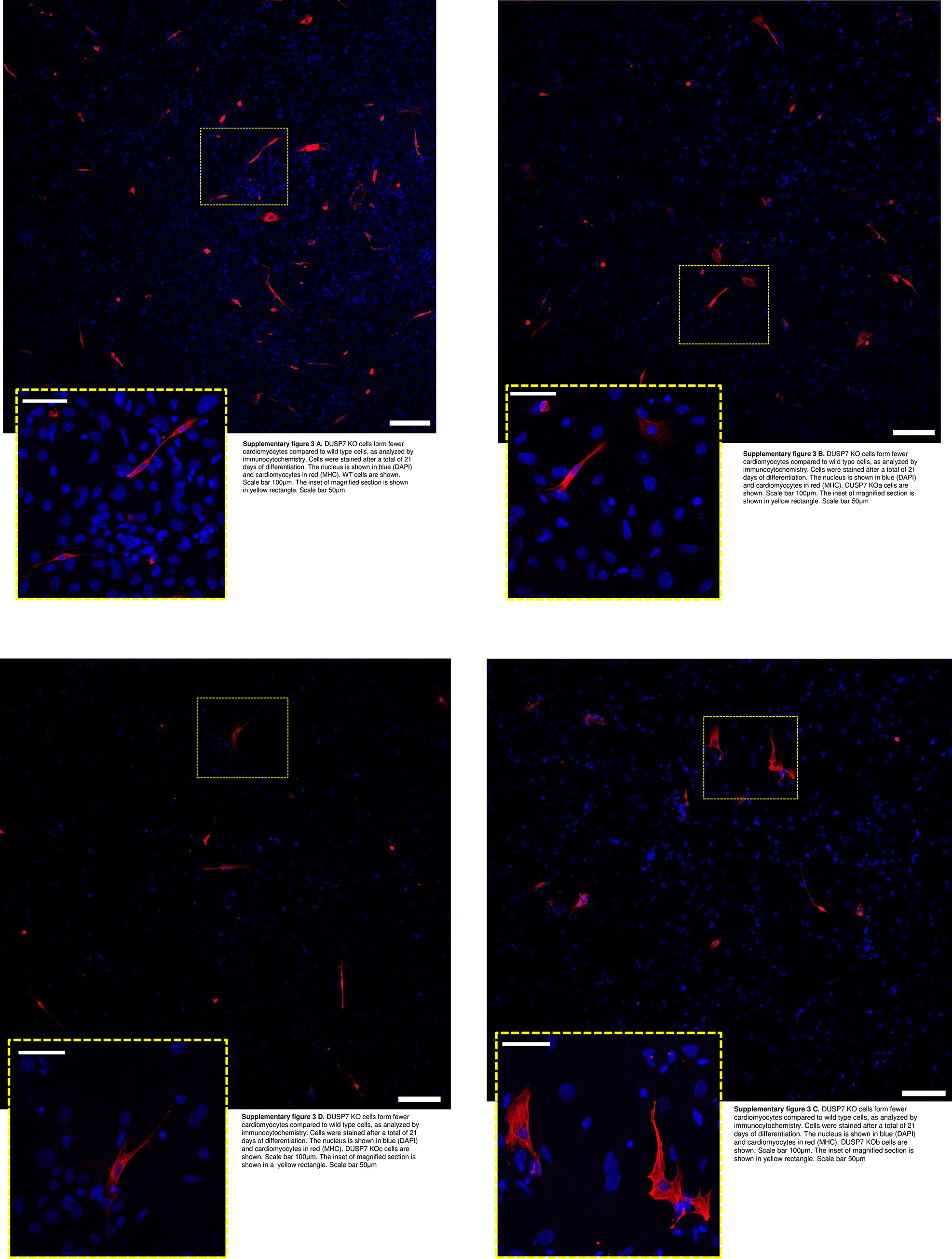

Supplement: S3 Fig — A: DUSP7 KO cells form fewer cardiomyocytes compared to wild type cells, as analyzed by immunocytochemistry–WT cells, B: DUSP7 KO cells form fewer cardiomyocytes compared to wild type cells, as analyzed by immunocytochemistry–DUSP7 KOa cells, C: DUSP7 KO cells form fewer cardiomyocytes compared to wild type cells, as analyzed by immunocytochemistry–DUSP7 KOb cells, D: DUSP7 KO cells form fewer cardiomyocytes compared to wild type cells, as analyzed by immunocytochemistry–DUSP7 KOc cells. (TIF) [file pone.0275860.s003.tif]

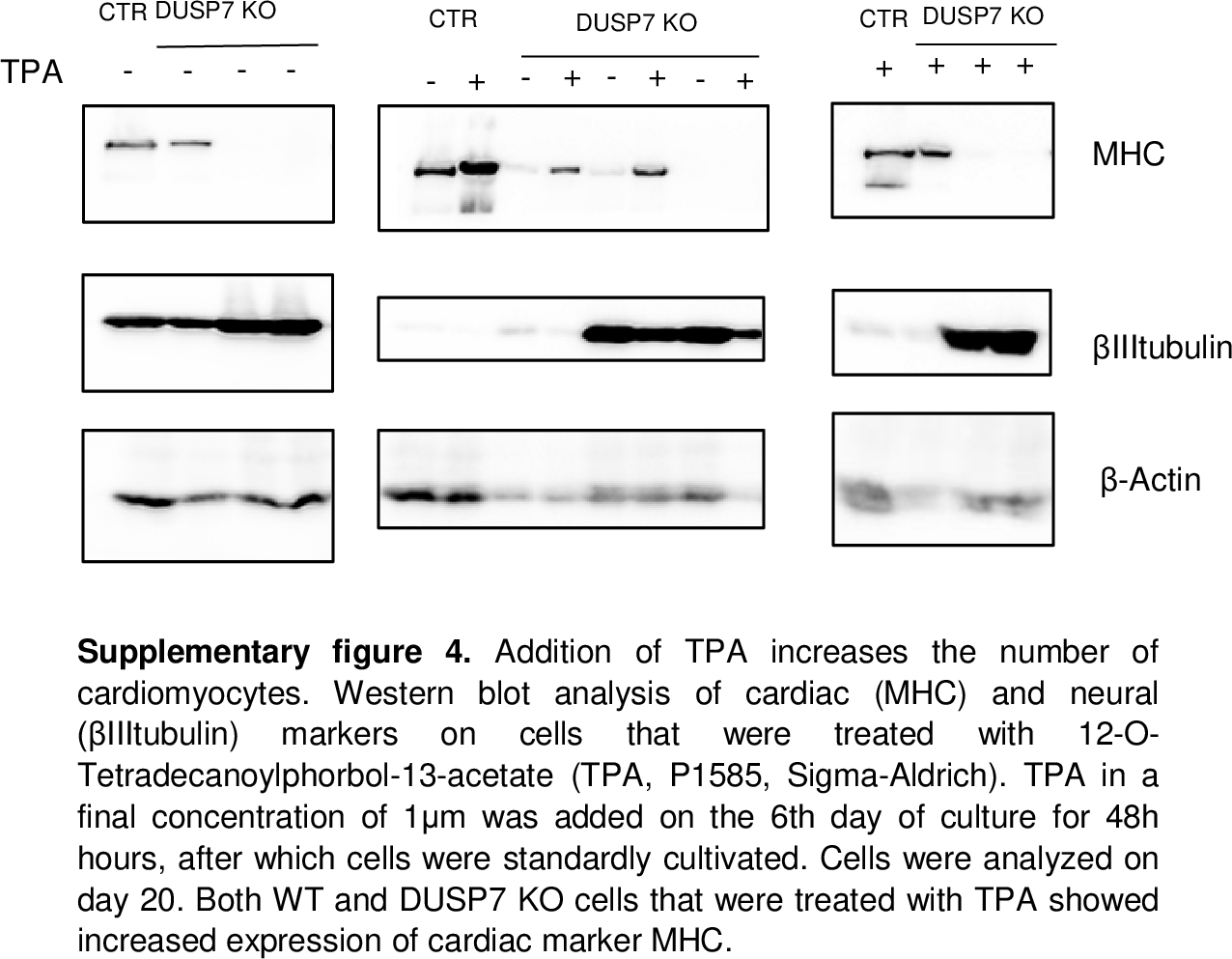

Supplement: S4 Fig — (TIF) [file pone.0275860.s004.tif]

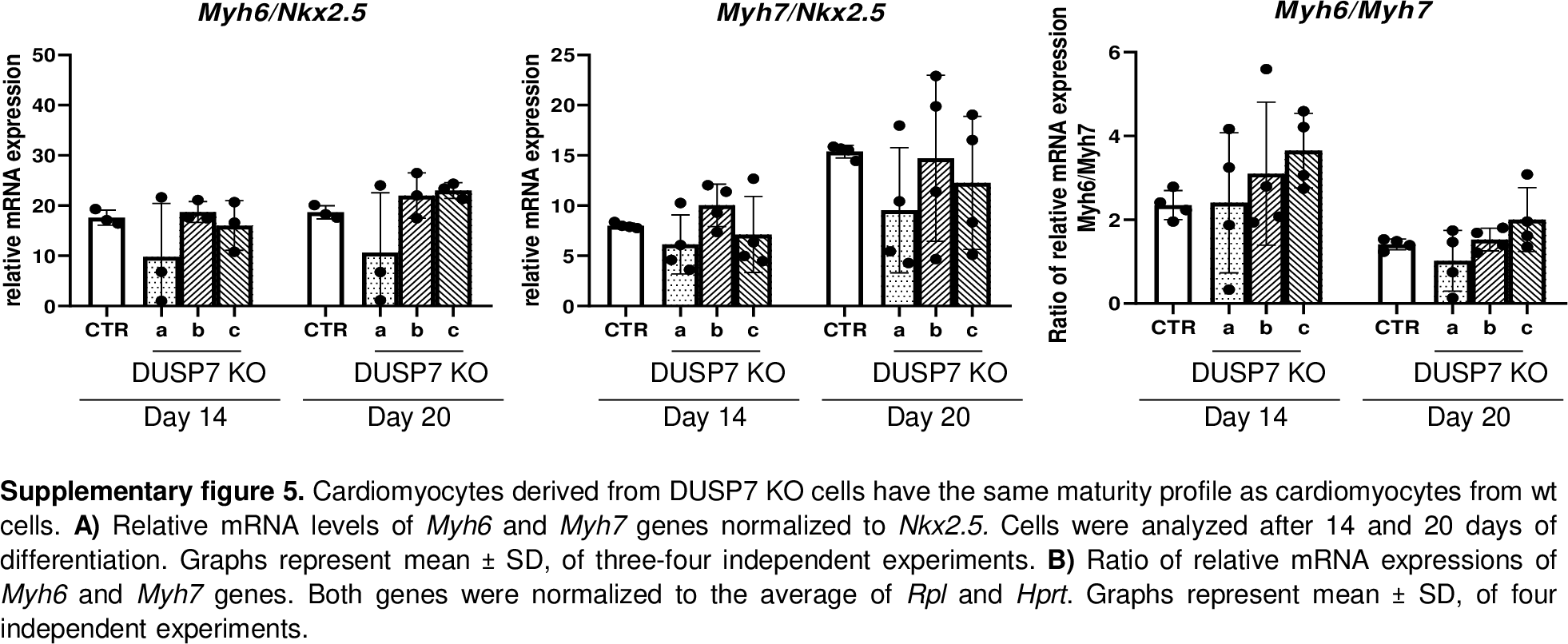

Supplement: S5 Fig — (TIF) [file pone.0275860.s005.tif]

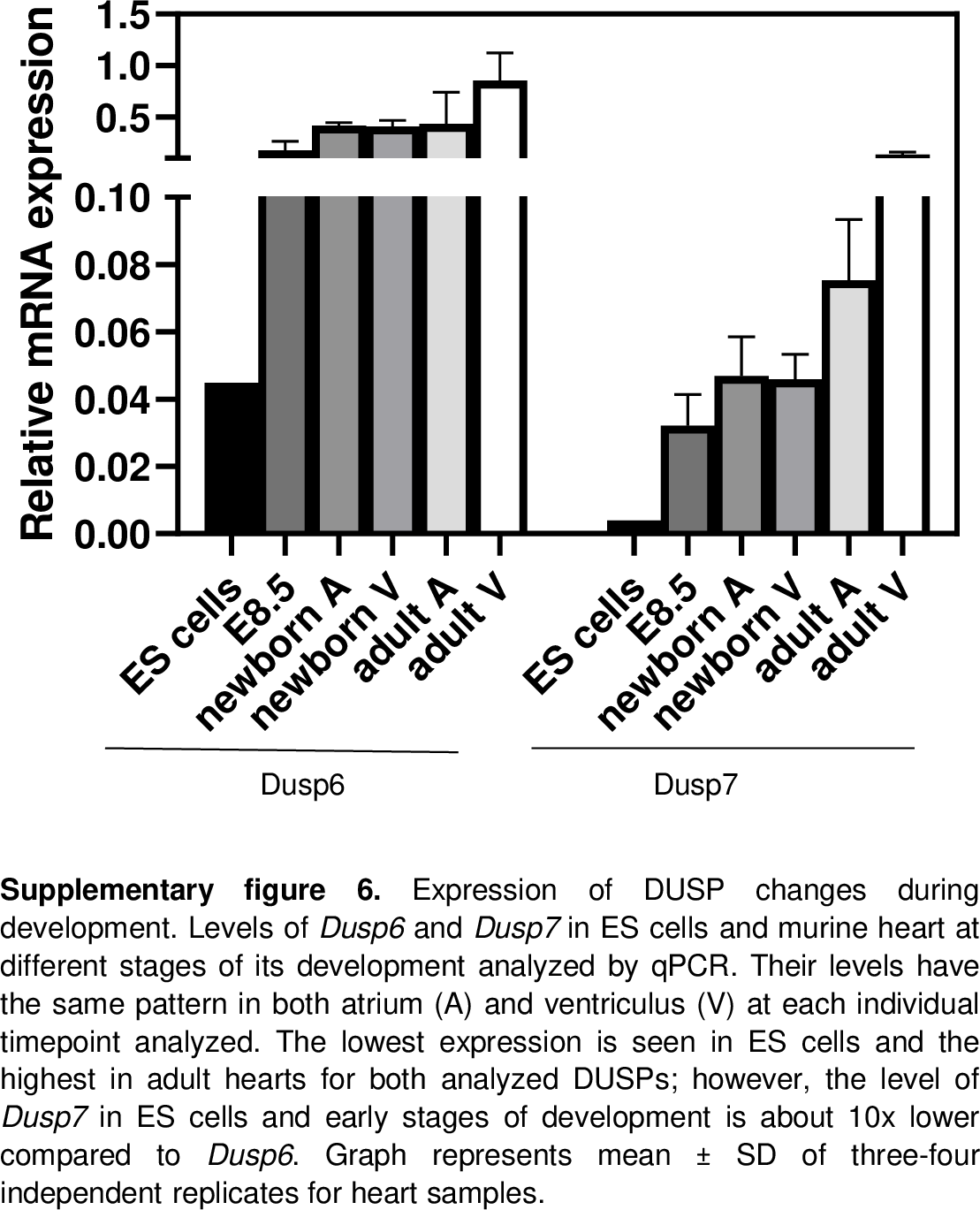

Supplement: S6 Fig — (TIF) [file pone.0275860.s006.tif]

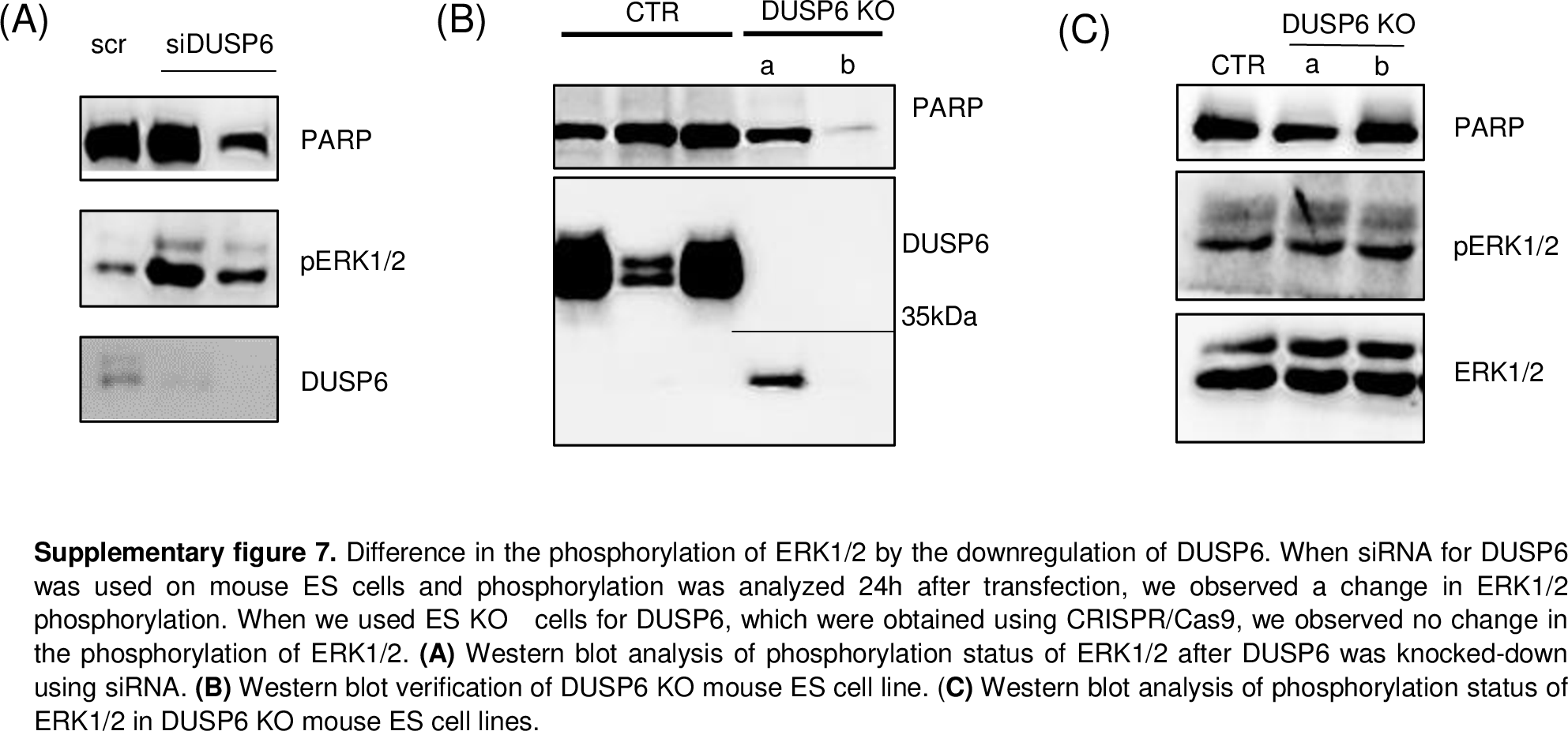

Supplement: S7 Fig — (TIF) [file pone.0275860.s007.tif]
