# Supplementary material for: Dual specificity phosphatase 7 drives the formation of cardiac mesoderm in mouse embryonic stem cells
Source: PLoS One. 2022 Oct 13;17(10):e0275860. doi: 10.1371/journal.pone.0275860 (PMC9560500; doi:10.1371/journal.pone.0275860)
Supplement: S1 Raw images — (PDF) [file pone.0275860.s008.pdf]

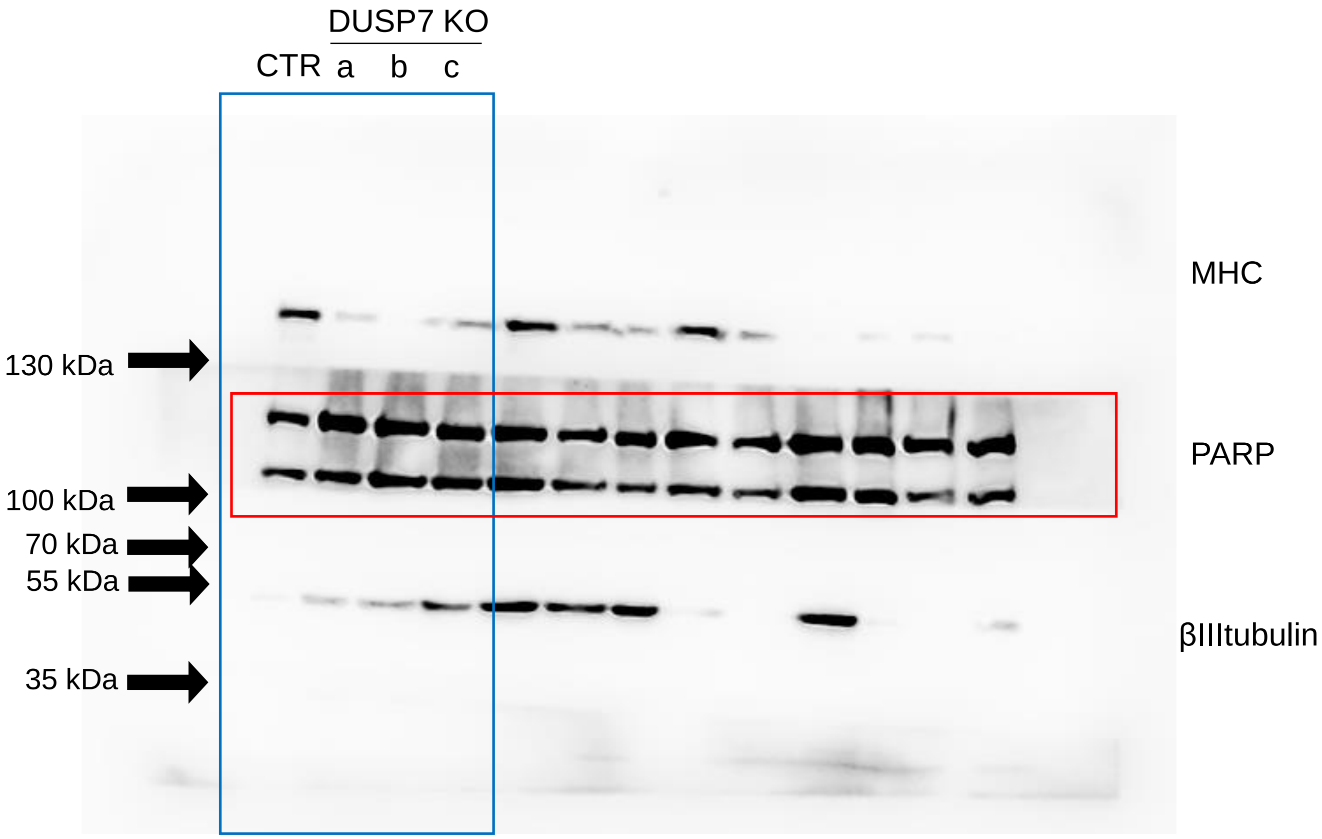

Unprocessed Western blot membranes with molecular mass marker related to Fig 4A. . Red rectangle marks specific bands. Blue rectangle marks relevant samples. Exposition time for all bands is 30s.

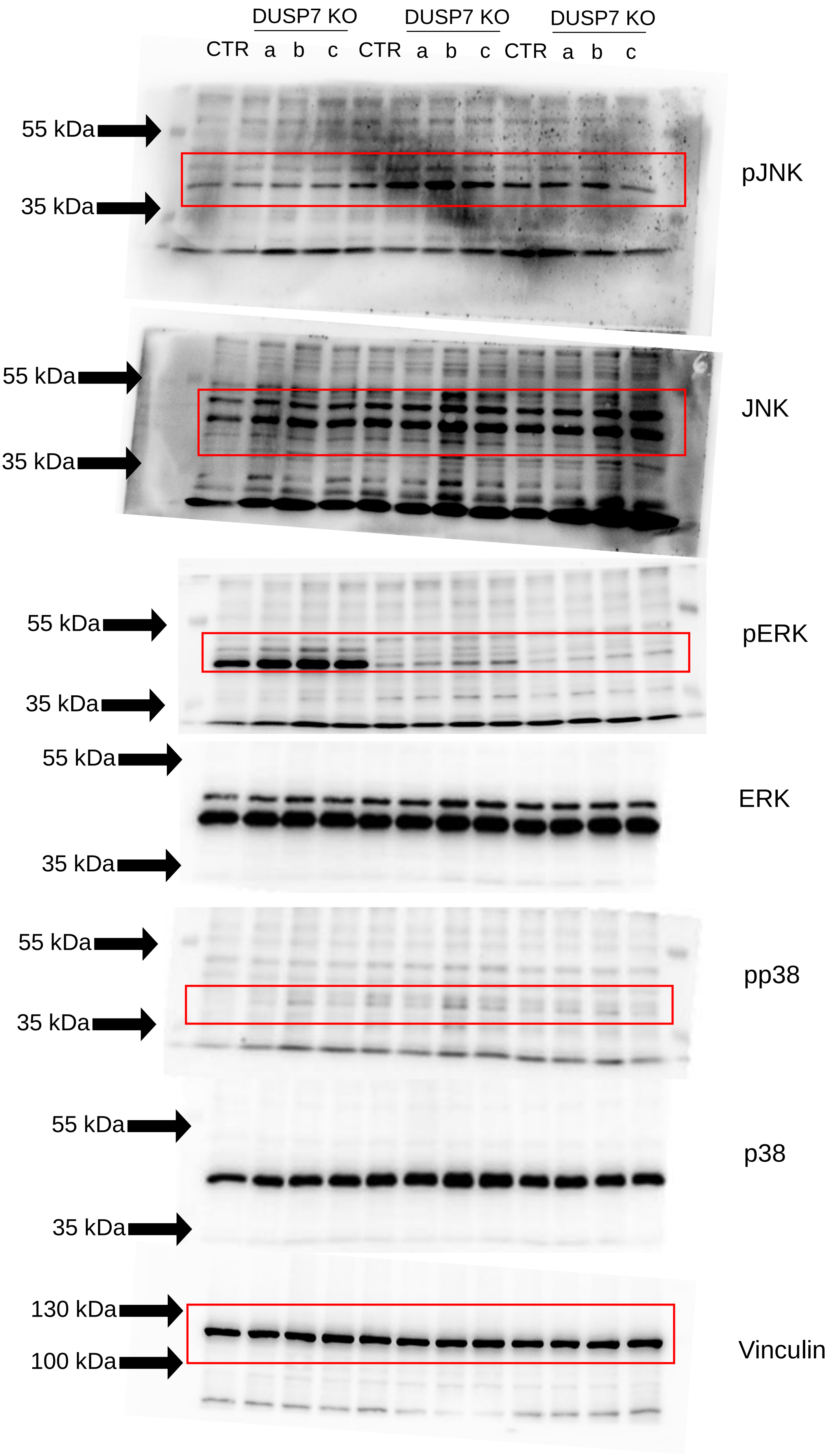

Unprocessed Western blot membranes with molecular mass marker related to Fig 6A. Red rectangle marks specific bands. Exposition time for Vinculin, ERK and p38 is 15s, for pERK and pp38 is 2min and for pJNK and JNK is 5min.

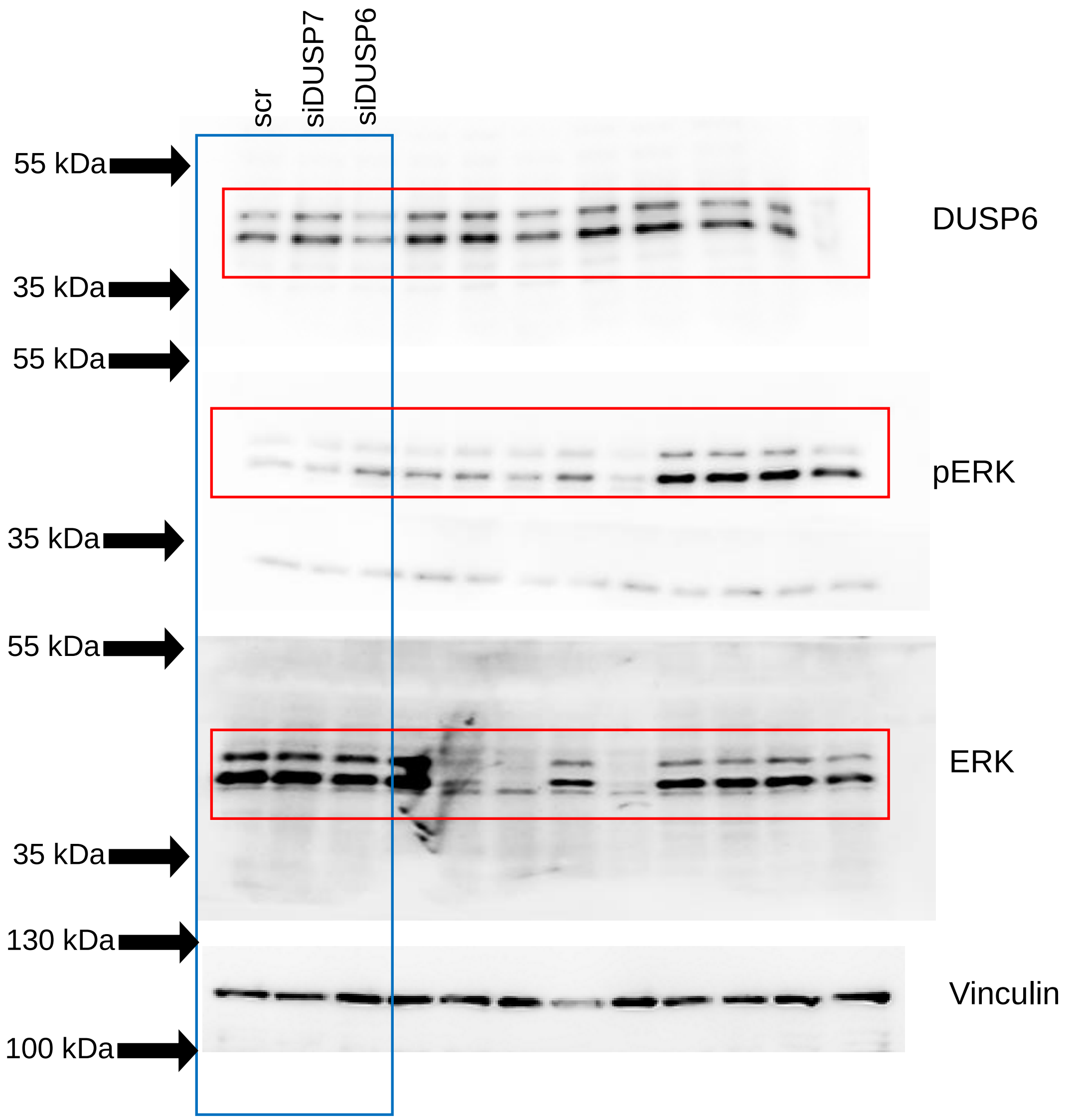

Unprocessed Western blot membranes with molecular mass marker related to Fig 6B. Blue rectangle marks relevant samples. Red rectangle marks specific bands. Exposition for vinculin and DUSP6 was 30s, 1min for pERK and 3min for ERK.

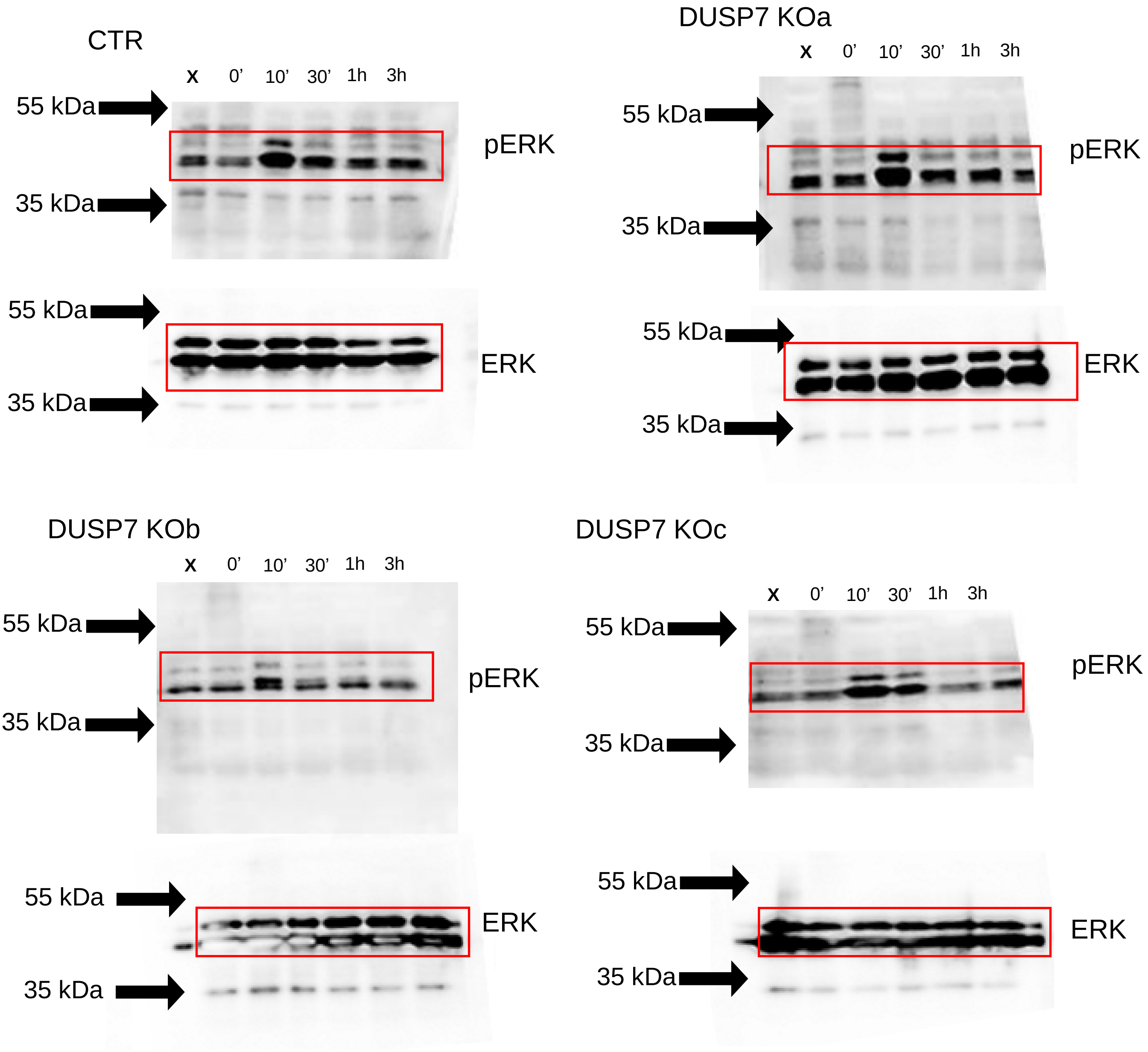

Unprocessed Western blot membranes with molecular mass marker related to Fig 6C. Red rectangle marks specific bands. Exposition time for ERK is 15s, for pERK 105s.

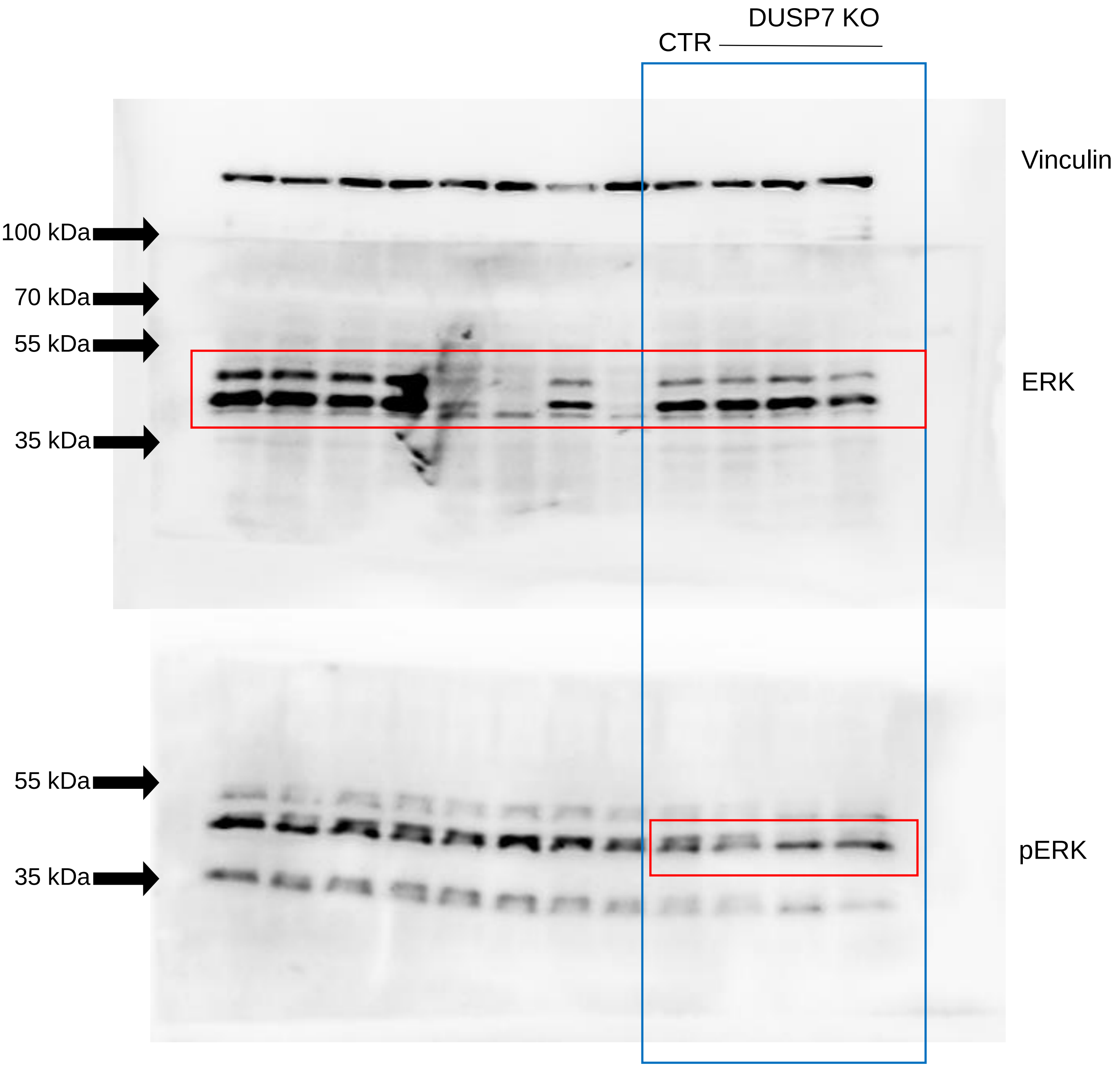

Unprocessed Western blot membranes with molecular mass marker related to Fig 7C. Blue rectangle marks relevant samples. Red rectangle marks specific bands. Exposition for all bands was 2min.

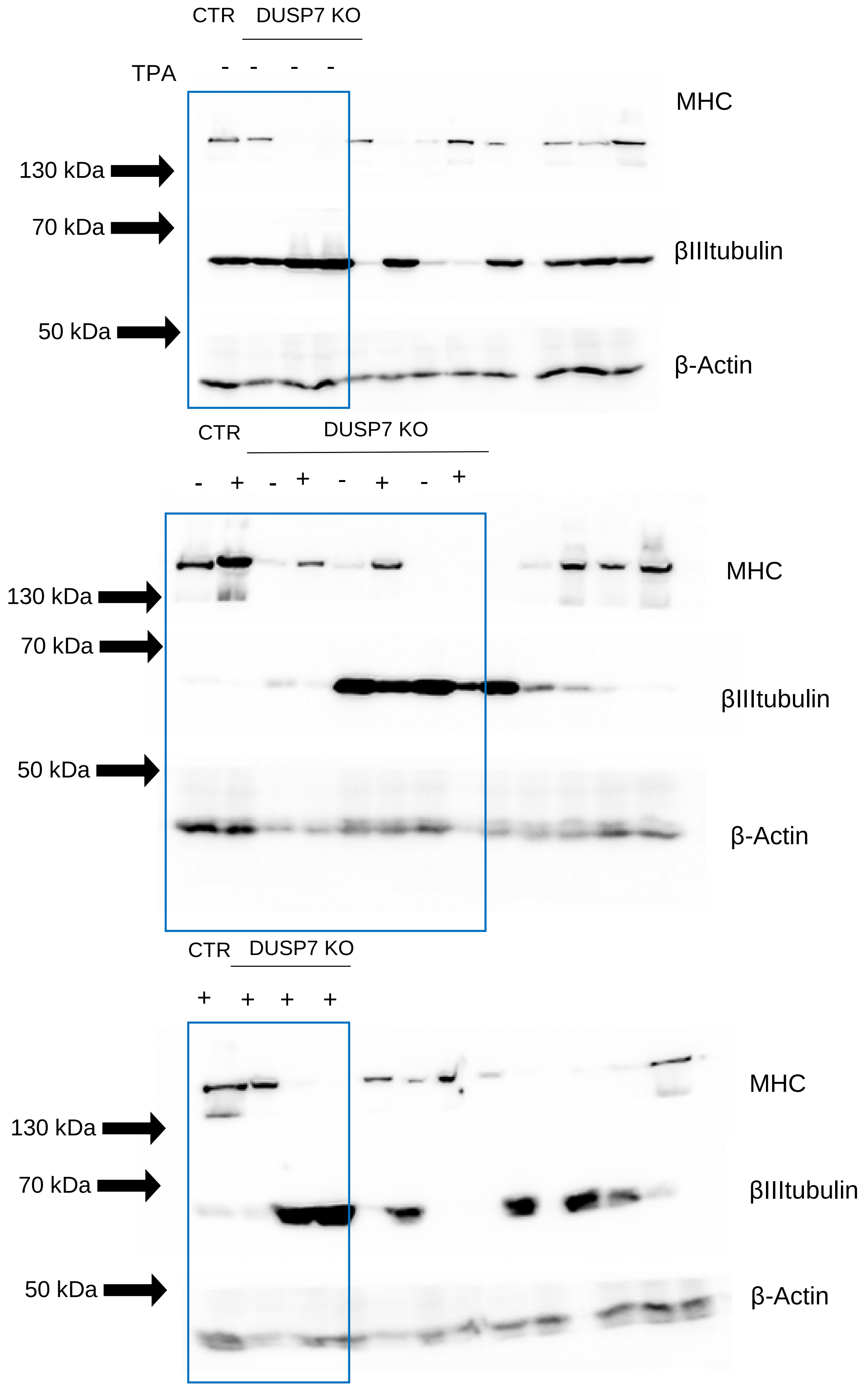

Unprocessed Western blot membranes with molecular mass marker related to Supplementary figure 4. Blue rectangle marks relevant samples. Exposition for all bands was 30s

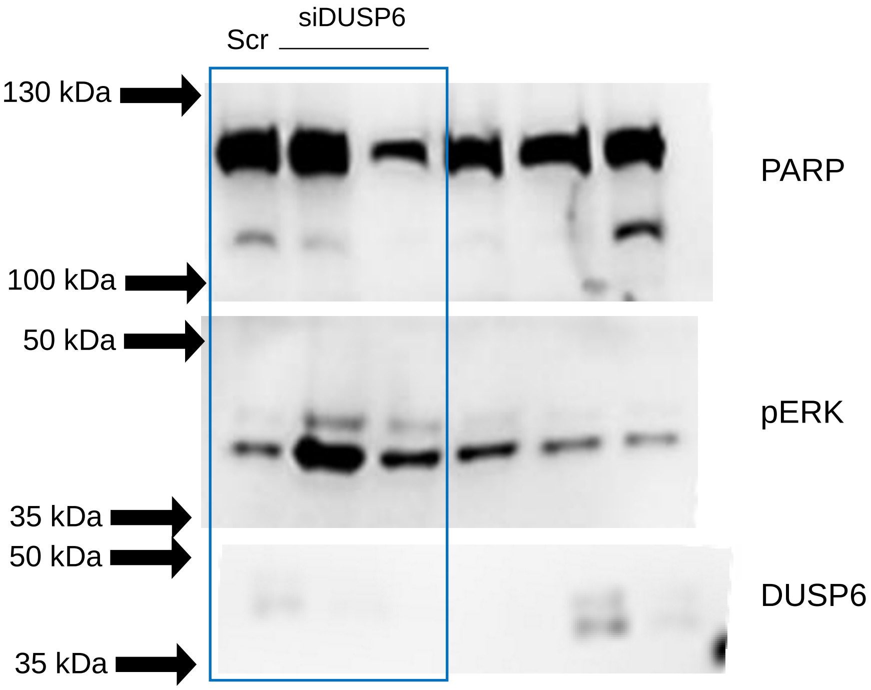

Unprocessed Western blot membranes with molecular mass marker related to Supplementary figure 7A. Blue rectangle marks relevant samples. Exposition for all bands was 30s

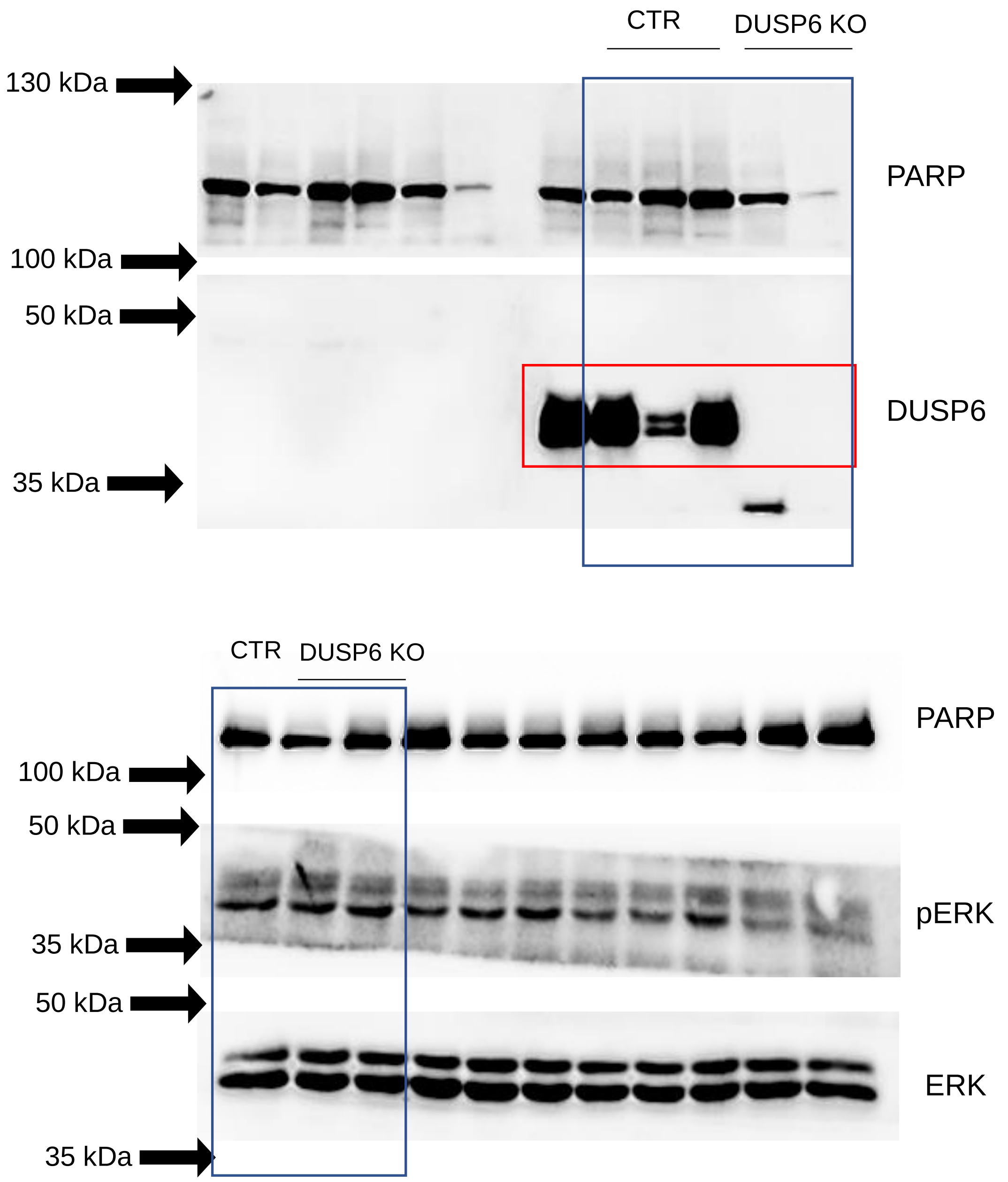

Unprocessed Western blot membranes with molecular mass marker related to Supplementary figure 7B and 7C. Blue rectangle marks relevant samples. Red rectangle marks specific bands. Exposition for PARP, ERK and DUSP6 was 30s, exposition for pERK was 3min.
