# Supplementary material for: Dual specificity phosphatase 7 drives the formation of cardiac mesoderm in mouse embryonic stem cells
Source: PLoS One. 2022 Oct 13;17(10):e0275860. doi: 10.1371/journal.pone.0275860 (PMC9560500; doi:10.1371/journal.pone.0275860)
Supplement: Attachment — Submitted filename: Response to Reviewers.docx [file pone.0275860.s009.docx]

Dear editor and reviewers,

Thank you very much for your comments to our manuscript. Please find bellow our responses to individual raised points. We hope they will be to your satisfaction.

**Reviewer #1:**

**The abstract should focus on the work done by the authors only and should not include any references.**

References, numbers from the abstract, have been removed.

**The cells were cultured for more than 40 passages, did they perform any karyotyping to determine if any chromosomal abnormalities were present in the cells after long shelf life?**

All experiments in which cells were differentiated as well as experiments based on which we were drawing conclusions on DUSP7’s effect on phosphorylation have been done in cells cultured 5-20 passages. The high passage was only used in experiment involving markers of pluripotency (Fig. 2D) to see if there will be some change in our KO cell lines in pluripotency even after longer shelf life. However, since we did not see any difference between the KO and WT cells, we did not investigate further changes that this kind of long-term cultivation could have. We have not performer karyotyping of the cells ourselves, but the used mouse embryonic stem cells line R1 (from which also all the KO cells lines were derived) is well established and defined cell line, which we have bought from ATCC (American Tissue Culture Collection).

**Why not perform overexpression of DUSP7 if you are talking about it in your discussion? I believe overexpression studies would have given strong support to your paper.**

Currently we are still working on determining the importance of DUSP7 as well as couple of other DUSPs in the development of early mesoderm and cardiomyocytes, where our preliminary results point to their importance in different stages of development. In that context, we agree that it would be very interesting to investigate the overexpression of DUSP7. However, this would be more interesting when it comes to pinpointing the importance of DUSPs in different stages, developmental intervals, than overall general effect that DUSP7 has in *in vitro* development from mouse ES cells, which we report in this paper.

**In Figures 1B and 1D, for example, the font is too small and some of the labels are not legible.**

We have exported pictures in high resolution and hope that this solves the issue of the labels not being legible.

**The quality of Figure 3 is very poor, either improve the resolution or substitute for the supplemental immunostaining pictures.**

We have exported the figures in high resolution (330dpi) which we hope resolves the issue. The pictures of embryoid bodies in Figure 3 were taken in hanging drops on stereomicroscope which does not provide very good quality of magnification. Furthermore, embryoid bodies are floating 3D objects that are difficult to focus on. Their pictures were used here more as an illustration to the graph next to them which shows the size of embryoid bodies on day 5. We cannot substitute them with immunostaining pictures from supplements, since those are taken on day 20 and have been cultured as adherent cells for 15 days. We hope that the quality in which they are now exported is acceptable.

**Figure 5 needs to show a cardiomyocyte-specific marker/antibody.**

We have changed the description of this figure, which now states that MF20 was used antibody instead of MHC. As stated in the material and methods (table 2) we used clone MF20 from the Developmental Studies Hybridoma Bank, which is specific for myosin heavy chain 6 and 7, which are considered as cardiomyocyte-specific markers.

**Reviewer #2:**

**Typo errors need to be checked.**

**a. In the abstract, there are some numerical numbers in each line (21 -31). It doesn’t make any meaning.**

This has been addressed and the numbers have been erased

**b. In section 3.5, DUSP7 is mentioned in small/capital letters. Please make it uniform throughout the manuscript.**

We call our cell lines DUSP7 KOa/b/c and generally when speaking about DUSP7, to have it more uniformed, we treat it as speaking about the mouse protein rather than gene and therefore write it all in capital letters. However, in case of section 3.5 as well as when its mentioned in Table 1 we are explicitly talking about measuring the gene expression of DUSP7 and therefore we used the the nomenclature which is used for mouse genes – italicized, with only the first letter in upper-case. We had mistaken in this regard in section 2.5 and Figure 1 which has been corrected.

**In fig. 5A, the scale bar was not visible clearly.**

We modified scale bar in fig. 5A and added it (Scale bar represents 100um) into the description of fig.5.

**The resolution of the figures is not good. Importantly the figure.1 text is hard to read. Please provide high quality figures.**

We have exported figures in high quality (330 dpi) which we hope resolves the issue.
